# Topographically Resolved Macular Pigment Optical Density Is Associated with Disease Progression in Macular Telangiectasia Type 2

**DOI:** 10.1016/j.xops.2026.101326

**Published:** 2026-07-15

**Authors:** Kristin Raming, Marie-Dominique Lewerenz, Nele Steffens, Jose Luis Rodriguez Garcia, Frank G. Holz, Kristina Pfau, Lukas Goerdt

**Affiliations:** Department of Ophthalmology, University Hospital Bonn, Bonn, Germany

**Keywords:** Macular telangiectasia type 2, MacTel, Macular pigment, Fundus autofluorescence

## Abstract

**Purpose:**

To investigate differences in topographically resolved macular pigment optical density (MPOD) between macular telangiectasia type 2 (MacTel) stages and to analyze its association with disease progression.

**Design:**

Longitudinal cohort study.

**Subjects:**

Participants of the Natural History and Observation Registry study and the University Eye Hospital in Bonn.

**Methods:**

Participants underwent multimodal retinal imaging including spectral-domain OCT, two-wavelength autofluorescence (2WAF) imaging for MPOD, and color fundus photography. Disease stage (0–6) was graded according to Chew et al. Macular pigment optical density en face maps were generated from 2WAF images, aligned to the fovea, and averaged within ETDRS subfields. Spatial differences across stages were assessed using linear mixed-effects models. Progression was defined as stage increase during follow-up and analyzed using univariable and multivariable regression models to estimate odds ratios for progression.

**Main Outcome Measures:**

Differences in topographically resolved MPOD, and association with disease progression.

**Results:**

A total of 265 eyes from 220 patients with MacTel (mean age 62.6 ± 7.7 years; 60.5% female) were analyzed. Eyes were staged as follows: stage 0 (38 eyes), stage 1 (45), stage 2 (67), stage 3 (12), stage 4 (24), stage 5 (46), and stage 6 (33). En face maps demonstrated stage-dependent alterations in MPOD: early temporal depletion and a preserved nasal–inferior bridge of MP in stages 1 to 4, followed by progressive central and nasal loss and attenuation of the parafoveal ring. Macular pigment optical density differed significantly across stages in the central and inner ETDRS subfields (all *P* < 0.001, except superior inner subfield), but not in outer subfields. Among the 232 eyes (stages 0-5) included in progression analysis (mean follow-up 2.5 ± 3 years), progressing eyes showed more pronounced temporal and nasal MPOD depletion. Higher MPOD in the temporal inner subfield was associated with reduced progression risk (odds ratio 0.97, *P* = 0.006).

**Conclusions:**

Macular pigment optical density maps reveal spatial alterations in MacTel and demonstrate the likelihood of progress of quantitative MPOD measurements. These findings may help identify eyes that could benefit from early neuroprotective interventions before irreversible vision loss occurs.

**Financial Disclosure(s):**

Proprietary or commercial disclosure may be found in the Footnotes and Disclosures at the end of this article.

Macular telangiectasia type 2 (MacTel) is a slowly progressive neurodegenerative disease of the central macula characterized by secondary vascular alterations, photoreceptor loss, and progressive visual impairment.^1^ A systemic metabolic component involving dysregulation of serine metabolism and accumulation of neurotoxic deoxysphingolipids has been identified as a central element of its pathobiology.^1^^,^^2^ Loss of the ellipsoid zone (EZ) on OCT, which correlates well with impaired visual function, serves as a structural surrogate marker of photoreceptor degeneration.^3^ The recent US Food and Drug Administration's approval of the ENCELTO implant, a cell-based neuroprotective therapy shown to slow EZ loss, marks the first disease-modifying treatment option for MacTel.^4^ However, there is currently a dearth of biomarkers indicating eyes at risk for disease progression before irreversible vision loss occurs.

One of the hallmark findings in MacTel is the depletion of macular pigment (MP).^5^ Macular pigment comprises the essential xanthophylls lutein and zeaxanthin (Z), as well as lutein’s metabolite meso-Z.^6^^,^^7^ They partition into cellular membranes of Müller cells and photoreceptors and are, to a lesser extent, found in the retinal pigment epithelium.^8^, ^9^, ^10^, ^11^ Macular pigment depletion in MacTel characteristically begins temporally to the fovea and may progress toward a circumferential redistribution pattern at approximately 4°- 7° eccentricity.^5^^,^^12^ Whether these changes represent true central MP loss, intraretinal redistribution, or relative alterations compared to the surrounding retina remains incompletely understood.

Macular pigment can be quantified as MP optical density (MPOD) in vivo via two-wavelength autofluorescence (2WAF) (488-nm excitation and 514-nm excitation).^13^ Macular pigment optical density is defined as the log_10_ ratio of green-excited autofluorescence intensity to blue-excited autofluorescence, compared to a retinal area where no MP is expected (9° eccentricity), calculated for each pixel.^14^ In MacTel, reduced MPOD has been associated with EZ loss, microperimetric scotomas, and functional impairment,^15^ suggesting potential utility as a biomarker of disease severity and progression. However, prior studies evaluating MPOD across disease stages have yielded inconsistent results, likely reflecting limited spatial resolution, small cohort sizes, and methodological heterogeneity.^16^, ^17^, ^18^ In addition, commercially available software (Heidelberg Eye Explorer, Heidelberg Engineering) averages MPOD within concentric circles (1°, 2°, and 9°) centered on the manually defined fovea. This approach inherently assumes radial symmetry of retinal alterations. While appropriate for healthy eyes and diseases that follow the distribution of retinal cells,^19^ it fails to capture the asymmetric, temporally predominant MP abnormalities characteristic of MacTel. Consequently, most prior studies have relied on qualitative MPOD assessment or broadly averaged regional values, limiting reproducibility and obscuring disease-specific topographic patterns.

Recent advances in pixel-wise image analysis allow spatially resolved mapping of MPOD independent of symmetry assumptions and have improved the understanding of MP topography in aging and age-related macular degeneration.^20^^,^^21^

In this study, we systematically analyze stage-dependent differences in MPOD distribution and evaluate the prognostic value of topographically resolved MPOD for disease progression.

## Methods

### Study Population

In this retrospective longitudinal study, patients with MacTel enrolled in the MacTel Natural History and Observation Registry Study^22^ at the Department of Ophthalmology, University Hospital Bonn, Germany, were analyzed.

The study was approved by the Medical Faculty, University of Bonn ethics committee (reference number 124/05) and adhered to the tenets of the Declaration of Helsinki. All participants provided written informed consent before study inclusion.

The inclusion criteria were a confirmed diagnosis of MacTel and clear optic media to allow high-resolution retinal imaging. The exclusion criteria were any confounding conditions, such as but not limited to age-related macular degeneration, central serous chorioretinopathy, uveitic diseases, severe cataracts impacting retinal imaging, and refractive errors higher or lower than ± 6 diopters. If both eyes of a participant were eligible, both eyes were included. In addition, 10 eyes of 10 healthy participants were included to allow a graphical comparison with early MacTel patients.

### Image Acquisition

All patients underwent a complete ophthalmologic examination, including slit lamp biomicroscopy and funduscopy after pupil dilatation with 0.5% tropicamide and 2.5% phenylephrine eye drops. Retinal imaging was performed using an investigational Spectralis HRA2 + OCT device (Heidelberg Engineering). The imaging protocol included combined near-infrared reflectance imaging and spectral-domain OCT (15° × 10°, 97 B-scans, high-resolution mode, automatic real-time averaging >9) and 2WAF (excitation wavelengths 488 nm and 514 nm; 30° × 30° scan field centered on the fovea). Color fundus photographs were obtained using the Zeiss Clarus 700 (Carl Zeiss Meditec).

### Disease Progression

Disease stages for all eyes were assessed using the multimodal imaging-based staging system (stages 0-6) by Chew et al^23^ by one experienced reading center–trained grader (L.G.). Disease progression was defined as an increase in MacTel stage according to the classification by Chew et al during follow-up. Macular pigment optical density was assessed at baseline only, and its association with disease progression was evaluated. Eyes were classified as either stable or progressing based on the presence or absence of stage increase during follow-up.

### Image Processing and Map Creation for MPOD Analysis

All OCT and 2WAF data were exported using the .XML format and were postprocessed using custom FiJi tools (freely available at https://sites.imagej.net/CreativeComputation/).^24^ For each OCT volume, the fovea was manually defined as the highest point of the inward rise of the external limiting membrane, where cone photoreceptors are at their longest,^25^ using the “Find_Fovea_OCT” plug-in. In cases in which the rise of the external limiting membrane was not visible, the lowest point of the foveal valley was selected.^26^ Two-wavelength autofluorescence images were postprocessed using “MPOD_XML_Reader,” which creates one 30° × 30° *en face* MPOD image per eye. Subsequently, MPOD levels were averaged within an ETDRS grid subfields per eye using “Grids_OCT.”^27^ To create en face MPOD maps, images of each disease group and healthy participants separately were averaged, using the foveal center and the optic nerve head as references to create stage-specific MPOD maps (“BatchStandardRetina”). Using the same approach, en face maps for stable and progressing eyes were created. In addition, to compare stable and progressing eyes more closely, we calculated pixel-level z-score maps for each group, using the following equation:

z=(x–μ)/σ

where z is the z-score, x is the value of the group being evaluated, μ is the mean of the entire population, and σ the standard deviation of the entire population.

### Statistical Analysis

Statistical analysis was performed using MatLab 2023b (The MathWorks, Inc). Parameters were summarized using means and standard deviations for normally distributed continuous variables and frequency distributions with percentages for categorical variables. Spatial MPOD differences across MacTel disease stages were analyzed using linear mixed-effects models to account for age and repeated measurements within eyes and patients. Eyes staged 0 to 5 were included in the progression analysis. Univariate regression was used to compare MPOD between stable and progressing eyes on an ETDRS subfield level. Subfields with significant differences were included in a multivariable regression model to estimate odds ratios (ORs) for disease progression per 0.01-unit increase in MPOD. Baseline disease stage and follow-up duration were included as covariates to adjust for differences in initial disease severity and observation time. A *P* value < 0.05 (two-sided) was considered significant. The subgroup of 10 healthy participants was not included in the statistical analysis, but is shown for graphical comparison.

## Results

### Cohort Characteristics

Of the 401 enrolled Natural History and Observation Registry participants at the Department of Ophthalmology at the University of Bonn, high-quality OCT, color fundus photographs, and 2WAF images were available in 265 eyes from 220 MacTel patients (mean age at baseline 62.6 years ± 7.7 years, 60.5% female) and were analyzed. Eyes were distributed across disease stages as follows: stage 0 (38 eyes, [14.3%]), stage 1 (45 eyes, [17.0%]), stage 2 (67 eyes, [25.3%]), stage 3 (12 eyes, [4.5%]), stage 4 (24 eyes, [9.1%]), stage 5 (46 eyes, [17.4%]), and stage 6 (33 eyes, [12.5%]) with overall comparable age. For graphical comparison, we included 10 eyes of 10 healthy control patients (mean age 44.6 ± 13.7 years, 8 female, 10 phakic). Table 1 summarizes the characteristics of the MacTel eyes.

**Table 1:** Cohort Characteristics and Disease Stage According to Chew et al^23^

Characteristic	All	Stage 0	Stage 1	Stage 2	Stage 3	Stage 4	Stage 5	Stage 6
Patients	220	34 15.5%	40 18.2%	53 24.1%	10 4.5%	20 9.1%	37 16.8%	26 11.8%
Eyes	265	38	45	67	12	24	46	33
Female	133	16	20	33	6	13	25	12
Age	62.6 ± 7.7	61.1 ± 11.2	62.7 ± 8.5	59.5 ± 9.9	65.0 ± 8.9	64.3 ± 7.4	64.7 ± 10.3	65.9 ± 11.1
Phakic	202	33	35	47	12	16	30	19

Demographics of MacTel patients with high-quality OCT, color fundus photographs, and two-wavelength autofluorescence available. Data are reported as number percentage of patients or as average ± standard deviation.

### Spatial MPOD Distribution across Different Disease Stages

**Figure 1:**
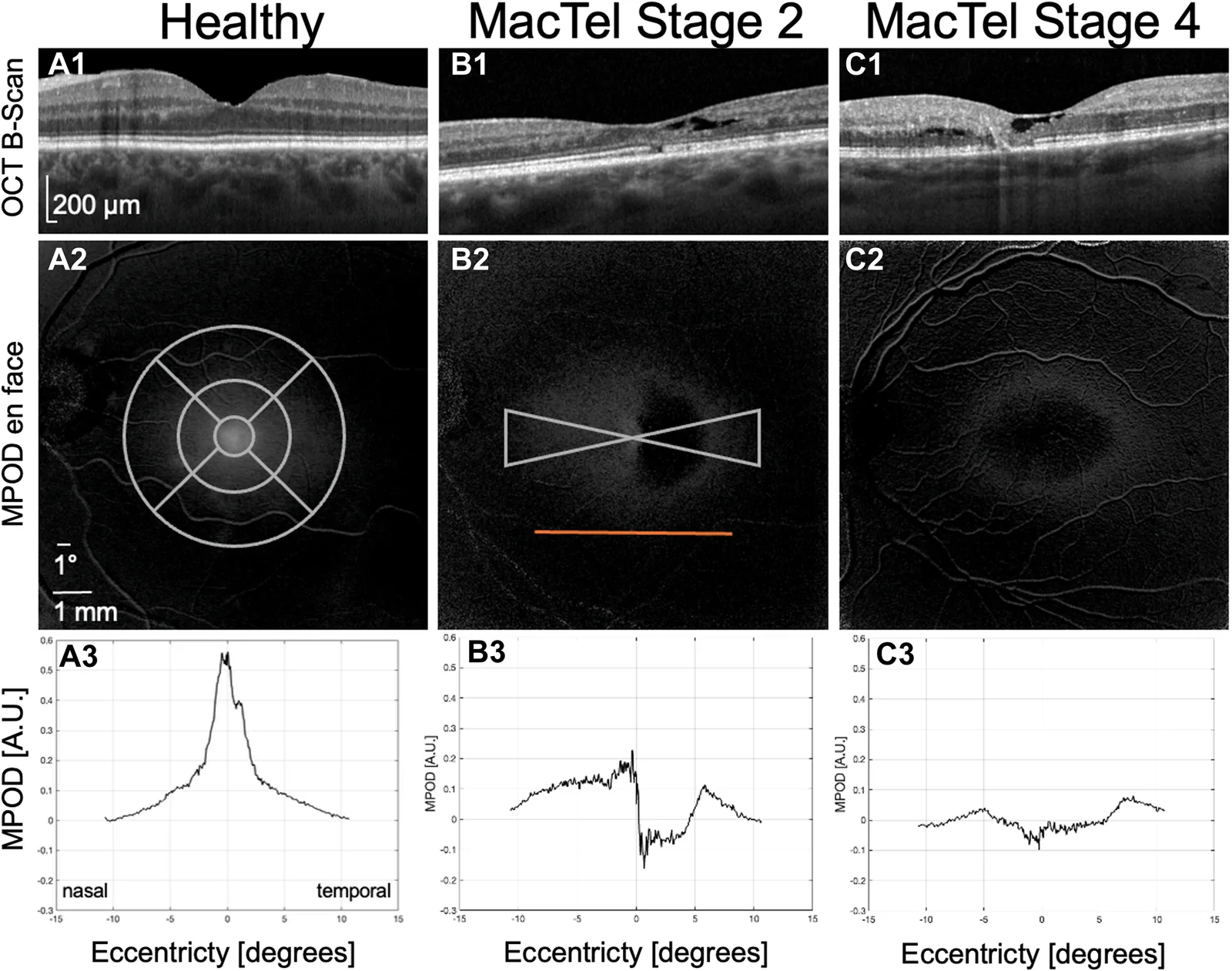
Macular pigment distribution in different MacTel disease stages. **A1**-**A3**: left eye of a 56-year-old healthy male. **B1**-**B3**: left eye of a 57-year-old female, MacTel stage 2. **C1**-**C3**: left eye of a 60-year-old male, MacTel stage 4. The horizontal extent of the foveal B-scans (**A1**, **B1**, **C1**) is represented by the orange line in **B2**. The gray-scale MPOD image in A2 shows a central peak inside the central subfield of the ETDRS grid. **B2** shows a temporally pronounced MPOD decrease, and **C2** shows an oval-shaped area of decreased MPOD. **A3**, **B3**, and **C3** show histograms of MPOD sampled from the bowtie depicted in B2. The histograms reproduce the en face shapes visible in **A2**, **B2**, and **C2**. MacTel = macular telangiectasia type 2; MPOD = macular pigment optical density.

**Figure 2:**
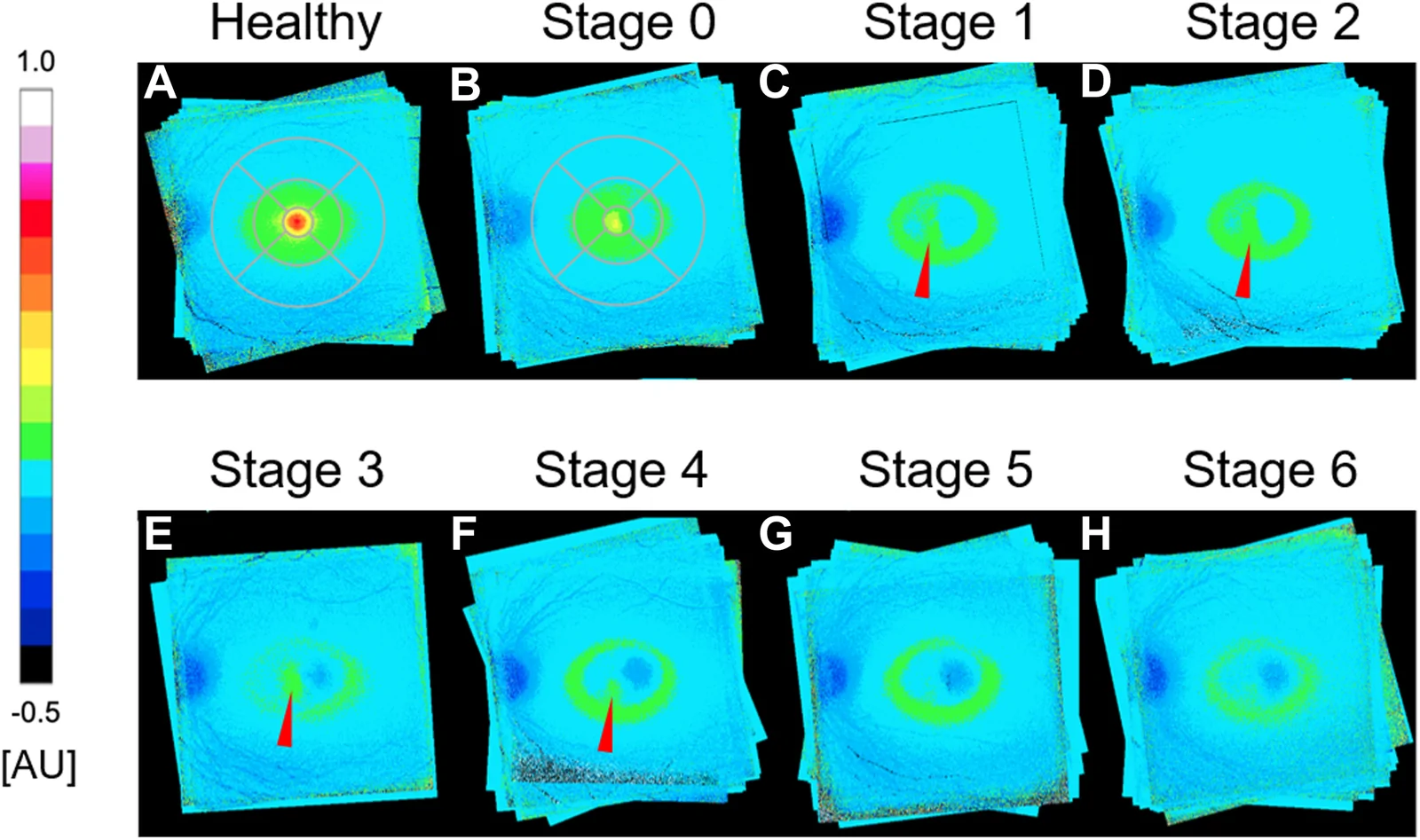
Spatial distribution pattern of MPOD across different MacTel stages. Pixel-wise MPOD maps in healthy eyes and MacTel disease stages 0 to 6. Color scale indicates MPOD values (AU). In healthy eyes (**A**), MPOD demonstrates a central peak with concentric decline toward the periphery. In early MacTel (stage 0, **B**), a focal temporal reduction of central pigment becomes apparent. With increasing disease stage (stages 1-4, **C**-**F**), the central peak disappears and temporal MPOD is reduced further, revealing an ellipse of MPOD at 4° × 7° eccentricity. Additionally, these stages maintain a nasal–inferior bridge of preserved MPOD (red arrowheads). In advanced stages (stages 5-6, **G** + **H**), this bridge disappears, and central MPOD is reduced further. AU = arbitrary units; MacTel = macular telangiectasia type 2; MPOD = macular pigment optical density.

Figure 1 exemplarily shows the OCT B-Scan, 2WAF en face MPOD images, and the MPOD histograms in a healthy eye, stage 2 MacTel, and stage 4 MacTel. Macular pigment optical density is temporally reduced in stage 2 (B2 and B3) and more globally reduced in stage 4 (C2 and C3). Pixel-wise MPOD maps demonstrated a stage-dependent characteristic alteration of MP distribution, see Figure 2. Compared to healthy eyes (A), stage 0 MacTel shows an initial temporal reduction of MP (B). Stages 1 and 2 (C + D) show similar MPOD distribution characterized by a preserved nasal–inferior bridge of MP (red arrowheads). In stages 3 and 4 (E + F), this bridge (red arrowheads) becomes less apparent due to further nasal and central depletion of MPOD. Stage 5 (G) shows marked central MP depletion, while stage 6 (H) demonstrates additional attenuation of the residual parafoveal ring.

### Spatial MPOD Distribution in ETDRS Subfields

As shown in Table 2, MPOD values differed significantly across disease stages within the central and inner ETDRS subfields (all *P* < 0.001), with the exception of the superior inner subfield (*P* = 0.065). No significant differences in MPOD were observed across MacTel stages in the outer ETDRS subfields (all *P* > 0.05).

**Table 2:** Spatial Macular Pigment Optical Density Values Comparison between MacTel Stages

ETDRS Subfield	All MacTel	Stage 0	Stage 1	Stage 2	Stage 3	Stage 4	Stage 5	Stage 6	*P* Value	Healthy
CS	0.063 ± 0.086	0.158 ± 0.134	0.052 ± 0.051	0.064 ± 0.074	0.066 ± 0.064	0.042 ± 0.045	0.026 ± 0.038	0.036 ± 0.078	**<0.001**	0.4 ± 0.17
TI	0.025 ± 0.034	0.054 ± 0.043	0.024 ± 0.030	0.032 ± 0.031	0.013 ± 0.021	0.010 ± 0.026	0.007 ± 0.017	0.016 ± 0.032	**<0.001**	0.131 ± 0.058
II	0.051 ± 0.031	0.071 ± 0.033	0.056 ± 0.027	0.057 ± 0.029	0.042 ± 0.026	0.039 ± 0.034	0.041 ± 0.032	0.037 ± 0.025	**<0.001**	0.110 ± 0.052
NI	0.061 ± 0.041	0.086 ± 0.043	0.063 ± 0.035	0.066 ± 0.037	0.049 ± 0.038	0.055 ± 0.037	0.051 ± 0.047	0.043 ± 0.032	**<0.001**	0.120 ± 0.065
SI	0.068 ± 0.038	0.080 ± 0.039	0.071 ± 0.035	0.072 ± 0.034	0.053 ± 0.043	0.073 ± 0.049	0.061 ± 0.040	0.052 ± 0.032	0.065	0.123 ± 0.050
TO	0.035 ± 0.023	0.029 ± 0.021	0.038 ± 0.023	0.034 ± 0.024	0.037 ± 0.013	0.032 ± 0.023	0.041 ± 0.024	0.036 ± 0.025	0.244	0.033 ± 0.016
IO	0.009 ± 0.012	0.008 ± 0.009	0.012 ± 0.015	0.011 ± 0.011	0.003 ± 0.006	0.005 ± 0.008	0.005 ± 0.009	0.009 ± 0.015	0.066	0.009 ± 0.009
NO	0.018 ± 0.019	0.021 ± 0.017	0.016 ± 0.022	0.018 ± 0.021	0.015 ± 0.013	0.022 ± 0.020	0.018 ± 0.022	0.014 ± 0.016	0.613	0.030 ± 0.027
SO	0.009 ± 0.012	0.006 ± 0.009	0.008 ± 0.013	0.008 ± 0.009	0.007 ± 0.010	0.012 ± 0.016	0.013 ± 0.031	0.009 ± 0.028	0.184	0.005 ± 0.055

MacTel = macular telangiectasia type 2; CS = central subfield; TI = temporal inner; II = inferior inner; NI = nasal inner; SI = superior inner; TO = temporal outer; IO = inferior outer; NO = nasal outer; SO = superior outer.

Significant *P* values are in bold font. Healthy eyes shown for comparative purposes, not included in statistical analysis.

### Spatial MPOD in Stable and Progressing Eyes

**Figure 3:**
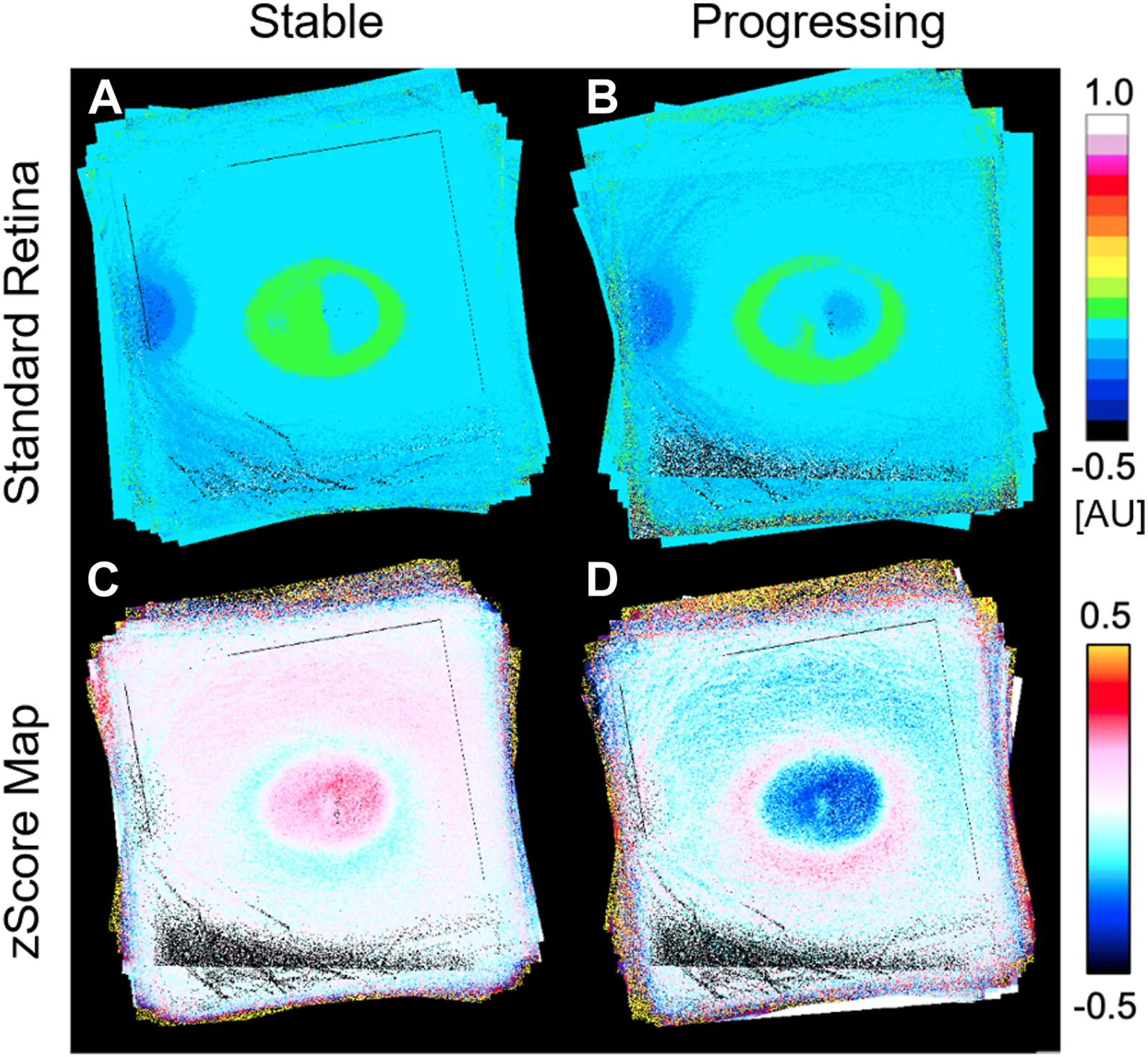
Macular pigment optical density distribution in stable and progressing MacTel eyes. Top row (**A** + **B**): standard pixel-wise MPOD maps. Bottom row (**C** + **D**): corresponding z-score maps normalized to the cohort mean. Stable eyes (**A**) demonstrate a preserved parafoveal ring with relatively maintained central values, whereas progressing eyes (**B**) show marked central depletion with expansion of low-density areas. Z-score mapping highlights early central reduction preceding visible structural changes and emphasizes spatial progression of macular pigment loss. AU = arbitrary units; MacTel = macular telangiectasia type 2; MPOD = macular pigment optical density.

A total of 232 eyes (stages 0 - 5) were available for progression analysis with a mean follow-up time of 2.5 ± 3 years. As shown in Figure 3, stable eyes show temporally reduced MPOD, whereas central and nasal areas are less affected by MPOD depletion. In progressing eyes, MPOD depletion is even more advanced temporally, and MPOD is also more reduced in nasal areas. Z-score maps show that MPOD in stable eyes is higher in the center and is surrounded by a barrier of comparatively lower MPOD. In progressing eyes, compared to the entire population, MPOD appears depleted centrally and surrounded by a barrier of higher MPOD.

Table 3 indicates that MPOD differed statistically between stable and progressing eyes in the central and temporal inner (both *P* < 0.001), inferior inner (*P* = 0.002), nasal inner (*P* = 0.035), and superior outer (*P* = 0.019) subfield.

**Table 3:** Macular Pigment Optical Density Differs between Stable and Progressive Eyes

ETDRS Subfield	Stable (*n* = 139)	Progressive (*n* = 93)	*P* Value
CS	0.077 ± 0.094	0.039 ± 0.061	**<0.001**
TI	0.032 ± 0.035	0.012 ± 0.026	**<0.001**
II	0.056 ± 0.030	0.043 ± 0.031	**0.002**
NI	0.065 ± 0.039	0.055 ± 0.042	**0.035**
SI	0.069 ± 0.037	0.066 ± 0.040	0.290
TO	0.035 ± 0.023	0.036 ± 0.022	0.684
IO	0.010 ± 0.012	0.007 ± 0.010	0.055
NO	0.018 ± 0.019	0.019 ± 0.022	0.714
SO	0.008 ± 0.010	0.011 ± 0.015	**0.019**

CS = central subfield; TI = temporal inner; II = inferior inner; NI = nasal inner; SI = superior inner; TO = temporal outer; IO = inferior outer; NO = nasal outer; SO = superior outer.

Significant *P* values are in bold font.

#### Spatial MPOD Associated with Disease Progression

As shown in Table 4, higher MPOD in the temporal inner ETDRS subfield decreases the risk for progression (OR 0.97 per 0.01-unit increase; 95% confidence interval (CI) 0.93 – 0.98; *P* = 0.006), whereas higher MPOD in the superior outer ETDRS subfield increases the risk for progression (OR 1.03 per 0.01-unit increase; 95% CI 1.01 – 1.04, *P* = 0.021). No significant association between MPOD in other ETDRS subfields and disease progression was observed.

**Table 4:** Macular Pigment Optical Density Associates with Disease Progression

ETDRS Subfield	OR	95% CI	*P* Value
Intercept	1.02	1.00–1.04	0.135
Age	1.00	1.00–1.00	0.079
CS	0.96	0.96–1.01	0.701
TI	0.97	0.93–0.98	**0.006**
II	1.00	0.99–1.00	0.184
NI	1.03	0.96–1.05	0.364
SO	1.03	1.01–1.04	**0.021**

MPOD = macular pigment optical density; OR = odds ratio; CI = confidence interval; CS = central subfield; TI = temporal inner; II = inferior inner; NI = nasal inner; SO = superior outer.

Significant *P* values are in bold font. Odds ratios are reported per 0.01-unit increase in MPOD.

## Discussion

In this retrospective study, we applied novel MPOD analysis tools enabling spatially resolved, topographic assessment of MP in MacTel. Both qualitative visualization and quantitative topographic analyses demonstrated significant differences across disease stages. The spatial pattern of MP loss mirrors the known spatiotemporal predilection of MacTel pathology, with pathologic changes occurring first temporally to the fovea.^28^ Macular pigment optical density en face maps revealed a preserved nasal–inferior “MP bridge” as a feature in intermediate to advanced disease stages. Preserved MPOD within the temporal inner ETDRS subfield was associated with a lower likelihood of disease progression.

The depletion of Müller glia plays a major role in MacTel. Lutein and Z are lipophilic and partition into cellular membranes.^6^^,^^7^ In healthy eyes, they are concentrated at the foveal center, defined as the area where cone photoreceptors and Müller glia are at their highest spatial densities,^29^ including the outer nuclear layer, with extensions into plexiform and nerve fiber layer.^9^^,^^30^, ^31^, ^32^ The relative contribution to total MP by Müller glia and photoreceptors in the foveola remains to be determined. As reviewed,^10^^,^^11^ the Müller glia hypothesis is supported by evidence from human eyes: loss of clinically detectable MP and histologically detectable Müller glia markers in donor eyes with MacTel, the detection of Müller glia markers and MP in surgically excised lamellar hole epiretinal membrane, and Müller glia abundance in the macula lutea, especially in retinal layers with high xanthophyll signal.^28^^,^^29^^,^^33^^,^^34^ Therefore, it appears reasonable to use MPOD as a surrogate marker for Müller glia.

Müller cells contribute to structural stabilization of the neurosensory retina, regulate metabolic exchange, and protect against oxidative stress, as well as provide neurotrophic support to photoreceptors.^35^^,^^36^ Additionally, MPOD correlates with photoreceptor density and structural integrity,^37^ and regions with preserved MP appear to exhibit slower photoreceptor degeneration.^15^

In MacTel, Helb et al^5^ described an initial MP depletion in the temporal parafoveal region, while Zeimer et al^38^ reported central pigment loss accompanied by perifoveal redistribution. Fundus autofluorescence studies by Wong et al^39^ further demonstrated that a mild increase in central autofluorescence may represent one of the earliest imaging signs of MacTel, even before other clinical or angiographic abnormalities become apparent. Esposti et al^17^ compared MPOD values of different disease stages, classified per the Gass and Blodi staging system,^9^ and did not find significant differences. However, their sample size was small, with the number of eyes per disease group ranging from 4 (stages 1 and 5) to 17 (stages 3 and 4). Chin et al^16^, investigating a small sample (n = 12) using heterochromatic flicker photometry for MPOD assessment, did not find differences between MacTel eyes in stages 1 and 2 compared to controls, but significant differences when comparing eyes staged 3 and 4 to controls. Müller et al^15^ described three qualitative MPOD distribution patterns and linked them to differences in EZ loss progression. Our research adds a quantitative component to these findings, showing that MPOD alterations indicate risk for disease progression. As preserved MP may reflect locally maintained Müller cell integrity, our findings support the hypothesis that localized preservation of MP may indicate a more stable retinal microenvironment and reduced susceptibility to disease progression in MacTel.

Our observations may, thus, have potential therapeutic implications. Current treatment strategies for MacTel, including the recently approved neuroprotective approaches,^4^ aim to slow disease progression rather than restore atrophic retinal structure. Consequently, therapeutic intervention may be most effective at stages when retinal architecture is still partially preserved. Regions with preserved MPOD may therefore represent retinal areas where the underlying cellular microenvironment remains sufficiently intact to respond to neuroprotective interventions. While partial recovery of the EZ has been reported in some cases,^40^^,^^41^ restoration of MP has not been demonstrated to date. This suggests that once MP is depleted, the associated cellular structures, particularly Müller cells, may already be irreversibly compromised. Consistent with this interpretation, previous studies have shown that after oral supplementation with lutein and Z, an increase in MP was detected only in retinal areas where pigment was present at baseline, whereas no increase was observed in regions where MP was already absent.^38^ Taken together, these findings support the hypothesis that early therapeutic intervention, before complete MP loss, may offer the greatest potential to modulate disease progression. Spatially resolved MPOD mapping may help to identify disease stages in which neuroprotective therapies could be most effective.

We observed a paradoxical association of superior outer MPOD with disease progression. However, this finding is likely of limited relevance. The ETDRS outer ring corresponds to an eccentricity of approximately 3 to 6 mm (≈9–19°), which lies largely outside the characteristic MacTel area typically described at approximately 4–7° eccentricity.^28^ As a result, the outer ETDRS sectors encompass retinal areas that are most likely spatially too large and too peripheral to adequately capture focal MP redistribution characteristic of MacTel. In addition, MPOD outside the central subfield and inner ETDRS ring is generally low, and differences may be statistically significant, yet clinically irrelevant. Consistent with this finding, the spatial MPOD maps (Fig 2) revealed no relevant alterations within the outer ring across different disease stages. The lack of consistent associations in the outer ETDRS subfields, therefore, underscores the strong regional specificity of MacTel-related MP alterations.

The MPOD maps presented in Figure 2 show a bridge of relatively preserved MPOD inferonasally inside the MacTel area. As discussed, structural alterations in MacTel occur first temporally to the fovea. To the best of the authors' knowledge, this is the first described pathologic finding that does not follow this typical distribution. However, hemispherical differences have been recognized in healthy maculae: Cellular populations and the retinal vasculature are generally denser in the superior compared to the inferior retina, which may render it more vulnerable to early neurodegenerative changes than the inferior hemisphere.^19^^,^^42^, ^43^, ^44^

Several limitations should be considered. First, no correction for lens status was applied, which may have influenced autofluorescence-based MPOD measurements. Second, the follow-up period was relatively short, limiting the ability to evaluate the impact of MPOD distribution on disease progression. Third, the presented study does not investigate the association between topographical MPOD, structural, and vascular metrics, which is warranted in future research.

Strengths of this study include the application of a novel, validated, and spatially resolved MPOD analysis approach and the large study cohort, allowing a detailed assessment of MP distribution across multiple disease stages.

In conclusion, we describe distinct disease stage-specific MPOD distribution maps and identify the prognostic relevance of quantitatively measured MPOD. Our results may help identify eyes that benefit most from neuroprotective treatment strategies before irreversible vision loss occurs. Future research should further investigate the relation between quantitative MPOD, structural, and microvascular changes to further elucidate drivers of MacTel pathology.
